# Fermentation-Guided Natural Products Isolation of a Grape Berry Triacylglyceride that Enhances Ethyl Ester Production

**DOI:** 10.3390/molecules23010152

**Published:** 2018-01-12

**Authors:** Christopher L. Blackford, Eric G. Dennis, Robert A. Keyzers, Claudia Schueuermann, Robert D. Trengove, Paul K. Boss

**Affiliations:** 1 Separation Science and Metabolomics Laboratory, 90 South Street, Murdoch University, Murdoch, WA 6150, Australia; blackford.christopher@gmail.com (C.L.B.); r.trengove@murdoch.edu.au (R.D.T.); 2 CSIRO Agriculture and Food, PMB 2, Glen Osmond, SA 5064, Australia; eric.dennis@ubc.ca (E.G.D.); schueuermann@bio.uni-frankfurt.de (C.S.); 3 School of Chemical and Physical Sciences, Victoria University of Wellington, P.O. Box 600, Wellington 6140, New Zealand; rob.keyzers@vuw.ac.nz

**Keywords:** grapes, wine, fractionation, ester, SPME-GCMS, chromatography, aroma, fermentation, triacylglycerides

## Abstract

A full understanding of the origin, formation and degradation of volatile compounds that contribute to wine aroma is required before wine style can be effectively managed. Fractionation of grapes represents a convenient and robust method to simplify the grape matrix to enhance our understanding of the grape contribution to volatile compound production during yeast fermentation. In this study, acetone extracts of both Riesling and Cabernet Sauvignon grape berries were fractionated and model wines produced by spiking aliquots of these grape fractions into model grape juice must and fermented. Non-targeted SPME-GCMS analyses of the wines showed that several medium chain fatty acid ethyl esters were more abundant in wines made by fermenting model musts spiked with certain fractions. Further fractionation of the non-polar fractions and fermentation of model must after addition of these fractions led to the identification of a mixture of polyunsaturated triacylglycerides that, when added to fermenting model must, increase the concentration of medium chain fatty acid ethyl esters in wines. Dosage-response fermentation studies with commercially-available trilinolein revealed that the concentration of medium chain fatty acid ethyl esters can be increased by the addition of this triacylglyceride to model musts. This work suggests that grape triacylglycerides can enhance the production of fermentation-derived ethyl esters and show that this fractionation method is effective in segregating precursors or factors involved in altering the concentration of fermentation volatiles.

## 1. Introduction

Volatile organic compounds in the wine matrix are responsible for determining the flavour and aroma of a wine and changes in concentrations of these volatile compounds will affect flavour and aroma attributes [1,2,3]. There is increasing evidence that grape components in a must can modulate the concentrations of not only varietal impact compounds [4,5,6], but also yeast-derived non-varietal compounds [7,8,9] in wines. Understanding the origins and processes involved in the formation or degradation of wine volatile compounds provides the potential to produce wines with specific flavour properties for targeted consumer groups. This knowledge will inform novel grape growing, wine making and ageing techniques that could allow such opportunities. 

To improve the understanding of the origins of volatile compounds in wine, grape-derived precursors to wine aroma compounds or grape-derived compounds that alter yeast volatile compound production need to be identified. As grapes and wine contain a very complex mix of compounds, fractionation of samples is a good method for simplifying these matrices for compound discovery. With a simplified matrix, examining the evolution of flavour and aroma compounds in a model system becomes less complex. Several methods have been employed for the fractionation of grape and wine material, including column chromatography, mixed layer counter-current chromatography (MLCCC) and solid-phase extraction (SPE) [10,11,12]. Several methods have targeted polar glycosidic precursors to monoterpenes and norisoprenoids in grapes and wine [13,14], as well as other fruits and vegetables [15]. Fractionation of volatile extracts of wines has also been shown to be an effective tool in understanding volatile compound contribution to wine aroma [16,17]. However, a robust, simple method for the fractionation of grape and wine matter of all polarities would be greatly beneficial to aid precursor identification for non-varietal aroma compounds, as well as varietal impact compounds. Such a method would also assist in identifying grape compounds that can indirectly affect wine volatile composition by altering yeast metabolism.

Pre-fractionation of extracts from organisms by polarity is a common technique used in the isolation of natural products to rapidly target fractions containing compounds of interest [18,19]. A method for crude extract fractionation, initially developed for the separation of marine sponge extracts, has great potential in the fractionation of grape and wine material [20]. This method involves passing aqueous dilutions of a crude organic extract through a poly(styrene-divinylbenzene) (PSDVB) resin. The process allows all non-polar and amphiphilic compounds of interest and of different polarities to be loaded onto one column prior to elution. Sequential stepped elution of the compounds from the loaded column with aqueous organic solvents of increasing organic content (usually acetone or methanol) allows fractionation of the adsorbed crude extract according to the polarity of the compounds in the extracts. 

This study aimed to test the effectiveness of fractionating organic extracts from grapes of the cultivars Riesling and Cabernet Sauvignon using the PSDVB loading process for aroma precursor studies. The utility of the method for obtaining useful fractionated and simplified grape material was determined by following the evolution of key aroma compounds from each fraction using fermentation experiments. Further fractionation resulted in the isolation of a fraction containing two isobaric polyunsaturated triacylglycerides; trilinolein and a triacylglyceride with oleoyl, linoleoyl and α-linolenoyl acyl chains that was able to induce higher ethyl ester production in model musts compared to controls. This was confirmed using model musts supplemented with trilinolein. The results demonstrate further evidence that the level of production of yeast-derived compounds in wine can be influenced by grape components.

## 2. Results

### 2.1. The PSDVB Loading Process Fractionates Grape Extracts by Polarity

The PSDVB loading method offers a convenient, repeatable method to fractionate organic extracts of marine sponges [20] and has been used previously on plant extracts [21]. This method was applied to organic grape berry extracts to test its usefulness in fractionating grape samples. Acetone extracts of Riesling and Cabernet Sauvignon whole berries were therefore partitioned on the PSDVB reversed-phase chromatographic material. Fractions of different polarities were obtained according to the acetone content of the aqueous solvent used for elution from the chromatographic material. Equal volumes of each fraction were dried to obtain non-volatile subsamples of each fraction. Headspace analysis by SPME-GCMS of aliquots of each dried fraction spiked in model wine revealed that typical wine volatile compounds were not present in any fraction above trace quantities (data not shown). The influence of the different grape fractions on wine aroma and volatile compound production during yeast fermentation was tested in small-scale model fermentations.

### 2.2. β-Damascenone and Terpenoids are More Abundant in Wines Made from More Polar Grape Fractions

**Figure 1:**
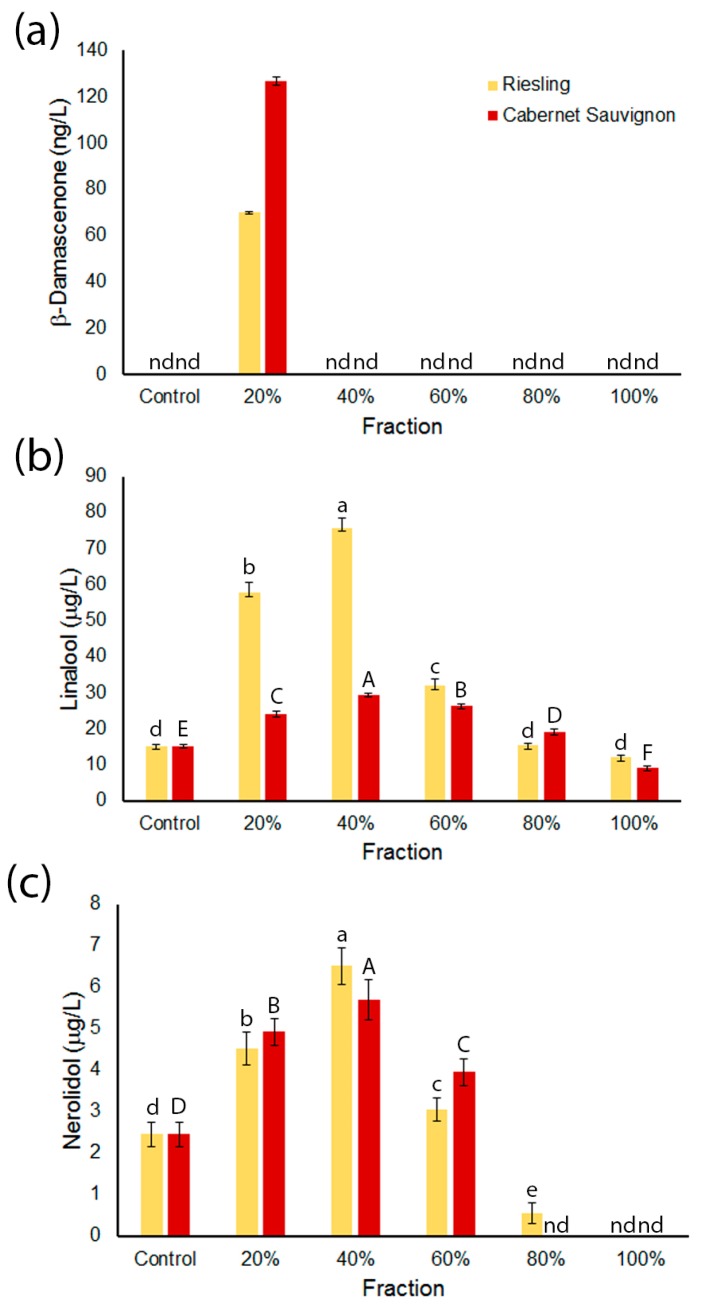
Concentrations of terpenoid compounds in wines produced after the addition of grape fractions. (**a**) *β*-Damascenone; (**b**) linalool and (**c**) nerolidol. Values represent the mean concentration of analyte (*n* = 3); error bars represent the standard error; and different letters denote significant differences between treatments at *p* < 0.05 using ANOVA followed by Duncan’s new multiple range test. ANOVA was not conducted for *β*-damascenone as it was only detected in one sample. Lower case = Riesling series. Upper case = Cabernet Sauvignon series.

To examine the effectiveness of the fractionation protocol, the concentrations of three compounds thought to be predominantly grape-derived were examined in the Riesling and Cabernet Sauvignon series of fermentations. These were the norisoprenoid *β*-damascenone (Figure 1a), the monoterpenoid linalool (Figure 1b) and the sesquiterpenoid nerolidol (Figure 1c). 

Observable and quantifiable amounts of *β*-damascenone could only be found in the wines made from the most polar fractions R20 (Riesling 20% acetone fraction) and CS20 (Cabernet Sauvignon 20% acetone fraction; Figure 1a). The pattern was more complex for linalool. In the Riesling series of fermentations, linalool concentrations were significantly higher in the wines made from R20, R40 and R60 compared to the control wines, with it being most abundant in R40 fermentations (Figure 1b). Wines made from less polar fractions did not appear to influence linalool production, as the concentrations in these wines were not significantly different from the control wines. For the Cabernet Sauvignon series, wines made from CS20–CS80 showed significantly higher concentrations of linalool compared to the control wines. The overall concentrations of linalool in these wines were lower than those in the Riesling series, especially comparing the wines made from 20% and 40% acetone fractions. 

Nerolidol, a sesquiterpenoid that has been suggested to impact wine aroma [22], showed similar trends in the wines made from the fractions purified from both grape varieties (Figure 1c). Nerolidol concentrations were significantly higher in wines made from the Riesling fractions R20, R40 and R60 and the CS20, CS40 and CS60 fractions from Cabernet Sauvignon when compared to control wines. In both series, nerolidol concentrations in wines made from the fractions obtained when 80% acetone and 100% acetone/ethyl acetate were used as the eluent were either at trace quantities or significantly lower than the control wines (Figure 1c).

### 2.3. Concentrations of Medium Chain Ethyl Esters and Medium Chain Fatty Acids in the Wines Are Influenced by Spiked Grape Fractions

**Figure 2:**
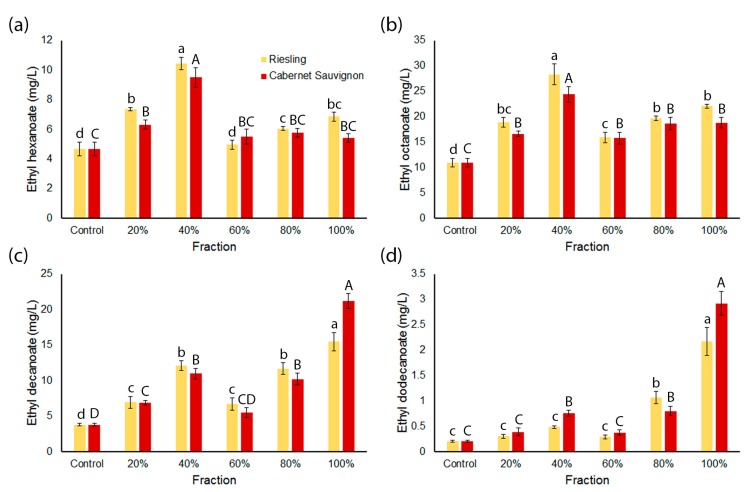
Concentrations of medium chain ethyl esters (MCEE) in wines fermented after addition of grape fractions. (**a**) Ethyl hexanoate; (**b**) ethyl octanoate; (**c**) ethyl decanoate and (**d**) ethyl dodecanoate. Values represent the mean concentration of analyte relative to the internal standard (*n* = 3); error bars represent the standard error; and different letters denote significant differences between treatments at *p* < 0.05 using ANOVA followed by Duncan’s new multiple range test. Lower case = Riesling series. Upper case = Cabernet Sauvignon series.

The concentrations of individual medium chain ethyl esters (MCEE) in spiked wines were significantly different compared to the control wines made with no grape content (Figure 2). In the Riesling series, the concentrations of ethyl hexanoate and ethyl octanoate were approximately 1.1–2.2-times and 1.5–3-times higher, respectively, in wines produced from model must spiked with a fraction of the grape extract compared to the controls, with the R40 wines showing the highest concentration (Figure 2a,b). Ethyl decanoate was approximately 2–4-times more concentrated in the Riesling fraction wines compared to controls, and ethyl dodecanoate was 1.5–10-times higher, with these esters being most abundant in the R100 wines (Figure 2c,d). In the Cabernet Sauvignon wines, the concentrations of ethyl hexanoate and ethyl octanoate were approximately 1.2–2-times and 1.5–2.2-times higher, respectively, in wines produced using grape fractions compared to control wines, with the CS40 wine containing the most (Figure 2a,b). Ethyl decanoate and ethyl dodecanoate showed respectively 1.5–5- and 1–15-fold increases in concentration compared to control wines, with these esters being most abundant in the CS80 and CS100 wines (Figure 2c,d). 

**Figure 3:**
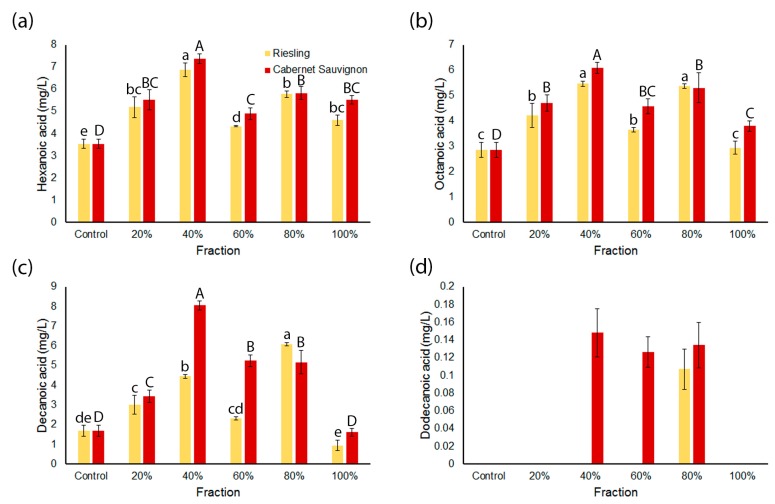
Concentrations of medium chain fatty acids (MCFA) in wines fermented after addition of grape fractions. (**a**) Hexanoic acid; (**b**) octanoic acid; (**c**) decanoic acid and (**d**) dodecanoic acid. Values represent the mean concentration of analyte relative to the internal standard (*n* = 3); error bars represent the standard error; and different letters denote significant differences between treatments at *p* < 0.05 using ANOVA followed by Duncan’s new multiple range test. Lower case = Riesling series. Upper case = Cabernet Sauvignon series.

Potential substrates for the production of these ethyl esters are the corresponding medium chain fatty acids (MCFA). While some differences were observed for the different wine varieties, there was a general trend that concentrations of the MCFA hexanoic acid, octanoic acid and decanoic acid were present in significantly higher concentrations in wines made with fractions obtained from 40% and 80% acetone eluents than the controls and wines produced with other fractions (Figure 3a–c). In the case of dodecanoic acid (Figure 3d), the Cabernet Sauvignon series showed similar trends to the other MCFA above, although it was only detected in the wines made after the addition of 40%, 60% and 80% acetone fractions. Dodecanoic acid was only detected in the wine made with an addition of the 80% acetone eluent for the Riesling series (Figure 3d).

### 2.4. Second Generation Fractionation of the CS100 Fractions on Diol Columns Results in the Identification of Triacylglycerides in a Fraction that Enhances MCEE Production during Fermentation

As the CS100 fraction was shown to increase the concentration of ethyl decanoate and ethyl dodecanoate apparently independent of the concentration of the corresponding carboxylic acid (Figure 2 and Figure 3), it was chosen for further fractionation. This was achieved by loading the two constituents of the CS100 fraction (i.e., the 100% acetone and 100% ethyl acetate fractions) onto separate diol columns in hexane, and the second generation of fractions was produced by eluting compounds adsorbed onto the columns with three column volumes of a series of 18 solvent mixtures of increasing polarity. 

The eluates were dried and 1 mg thereof used in a very small-scale fermentation process to assess the influence of the fractions on MCEE production. As the second round fractionation only yielded a small mass of material in each fraction, it was necessary to conduct these very small-scale fermentations and without replication to guide fraction selection for further identification. While it is unlikely that the conditions in such small fermentations completely mimic fermentation on an industrial scale, they were the best means to indicate the potential of the second round fractions to increase ethyl ester production relative to fermentations with no added fraction. The fermentations represented, essentially, a concentration of approximately 1 g/L of extract in the model must. The micro-fermentations were sampled seven days post-yeast inoculation when weight lost had stabilised. The relative concentrations of selected wine volatiles in the control wines and in the five wines that had the highest total MCEE concentrations are presented in Supplementary Figure S1. Wines made with fractions B1, A2, B16 or B15 had higher concentrations of ethyl hexanoate than the control wines (2.2–3.4-fold), and those made with the A10, B1, A2, B16 and B15 fractions had higher concentration of both ethyl octanoate (5–8.9-fold) and ethyl decanoate (9–51-fold) than the control wines. The wines made with supplements of fraction B16 or B15 also had increases in the concentration of ethyl dodecanoate relative to the control wines (1.4–1.8-fold). The volatile fatty acids were not elevated in the wines made from fraction-supplemented musts compared to the control wines, instead tending to have low to average concentrations, compared to the control wines, of each of the volatile fatty acids analysed (data not shown). The top candidate fraction (A10) was found by proton NMR to be contaminated with phthalate esters, which are common plasticisers that could have leached from multiple laboratory equipment sources. The fraction that resulted in the second highest concentrations of MCEE, B1, had a relatively “clean” proton spectrum and was therefore further investigated with MS and other NMR experiments. By means of 1D/2D NMR, HR-MS and LC-MS/MS experiments, the triacylglycerides TAG (18:2/18:2/18:2) and TAG (18:1/18:2/18:3) were confirmed in an approximate 1:1 ratio in fraction B1 (Appendix A).

### 2.5. Trilinolein Additions to Model Musts Influence MCEE in the Resulting Wines

**Figure 4:**
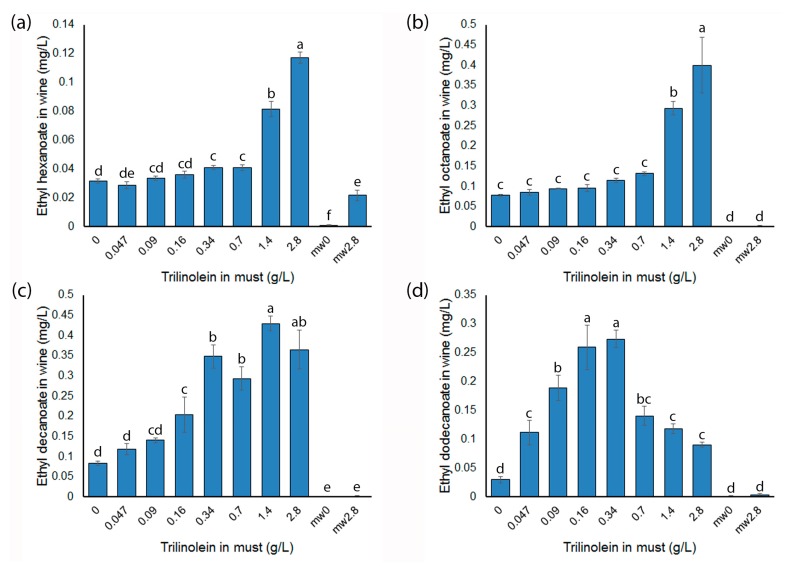
Concentrations of MCEE in wines prepared from model musts spiked with different concentrations of trilinolein. Values represent the mean concentration of analyte (*n* = 3); error bars are the standard error; and different letters denote significant differences between samples at *p* < 0.05 using ANOVA followed by Duncan’s new multiple range test. (**a**) Ethyl hexanoate; (**b**) ethyl octanoate; (**c**) ethyl decanoate; (**d**) ethyl dodecanoate.

Given that one of the triacylglycerides in fraction B1 was predicted to be trilinolein (TAG (18:2/18:2/18:2)), the ability of this compound to influence MCEE production was tested by adding different amounts to model must and fermenting with yeast strain EC1118. The fermentations completed in a similar timeframe and were harvested 14 days after inoculation. The concentrations of each of the four MCEE were significantly increased by the pre-fermentation addition of exogenous trilinolein (Figure 4). In the case of ethyl hexanoate, a significant increase in concentration was measured in wines made from musts with trilinolein concentrations above 0.34 g/L, with the greatest increase seen in wines made from musts with trilinolein concentrations of 2.8 g/L (Figure 4a). Ethyl hexanoate did not increase in wines made from musts with up to 0.16 g/L trilinolein relative to wines made without lipid supplements. Ethyl octanoate concentrations increased 3.3- and 4.2-fold in wines made from musts with 1.4 and 2.8 g/L trilinolein compared to wines made from control must, respectively(Figure 4b). Wines made from musts with trilinolein concentrations of 0.7 g/L or less had the same ethyl octanoate concentrations as wines made from un-supplemented must. The ethyl decanoate concentration of wines made from musts with trilinolein concentrations of less than 0.16 g/L was similar to that of wines made from un-supplemented musts. Each of the wines made from musts with trilinolein concentrations of 0.16 g/L or greater were found to have ethyl decanoate concentrations between 2.8- and 4-fold greater than those of wines made in control fermentations (Figure 4c). There was not a significant difference in ethyl decanoate concentration between wines made from musts with the two highest concentrations of trilinolein. Ethyl dodecanoate concentrations were higher than those of the control wines in all wines made from musts with trilinolein additions (Figure 4d). 

## 3. Discussion

To test if the fractionation strategy applied to whole grape acetone extracts could generate distinct fractions that differed in their ability to influence the production of wine volatiles, three compounds thought to be grape-derived were quantified in wines made by fermenting model grape juice medium (MGJM) containing an aliquot of each fraction. Figure 1 highlights that, overall, precursors or compounds that can stimulate the production of the aroma compounds *β*-damascenone, linalool and nerolidol during fermentation primarily segregate into more polar fractions (20% and 40% fractions) during fractionation. None of these compounds were present in the unfermented dried grape fractions. This suggests preferential segregation of precursors into these fractions. For *β*-damascenone and linalool, these observations are consistent with current hypotheses that polar terpenoid glycosides are precursors to monoterpenes and norisoprenoids [12,23,24] and so should separate into the more polar fractions. The results also indicate the presence of an unidentified precursor to the sesquiterpenoid nerolidol or other compounds that enhance nerolidol production during fermentation. An interesting observation was that wines made from CS100 contained significantly less linalool than the control wine (Figure 1b). While it is not surprising that the addition of the CS100 fraction produced less linalool in the wines than the more polar fractions, where linalool precursors are likely to elute, the observation that the CS100 wines produced significantly less linalool than the control wines was unexpected. As such, there may be compounds in this fraction that suppress linalool formation, which has been shown to occur during fermentation in the absence of grape material [25]. Alternatively, some of the linalool could be conjugated to or sequestered by another component in the wine, hence reducing the concentration of free linalool in the headspace. A similar phenomenon was seen with nerolidol concentrations in wines made from the 80% and 100% fractions from both grape varieties, whereby nerolidol production appears to be suppressed by components in those fractions or converted into another metabolite, be it volatile or non-volatile (Figure 1c). Nevertheless, the significantly higher abundance of these compounds in wines made from discrete fractions compared to the controls underlines the influence of grape components on their concentration in wine. The expected varietal differences in linalool concentrations were also observed with there being more linalool present in wines made from some of the Riesling fractions compared to those from Cabernet Sauvignon (Figure 1b). While not unexpected for known grape-derived wine volatile compounds, these observations establish this fractionation/fermentation method as a useful tool for identifying novel grape-dependent aroma compounds in wines.

The concentrations of the medium chain ethyl esters (MCEE) ethyl hexanoate, ethyl octanoate, ethyl decanoate and ethyl dodecanoate significantly differed in the wines made from the different grape fractions (Figure 2). These four MCEE were present in higher concentrations in wines made from 40% acetone fractions from both grape varieties compared to the control wines (except ethyl dodecanoate in Riesling). Concentrations were then lower in the wines made from 60% acetone fractions and, for ethyl decanoate and ethyl dodecanoate, then increased in wines made from 80% acetone and 100% acetone/ethyl acetate fractions. The relative increase in the concentrations of MCEE in wines made from 80% acetone and 100% acetone/ethyl acetate fractions compared to the control is reflected by length of the fatty acid chain where, in this case, dodecanoate > decanoate > octanoate > hexanoate (Figure 2). These results suggest that components in grapes contribute to the production of ethyl esters during fermentation and that these components can be segregated by polarity using the fractionation method employed in this study. A comparison of Figure 2 and Figure 3 may explain in part the trends in MCEE concentrations observed in wines made from 40%, 60% and 80% acetone fractions in both series, in particular ethyl hexanoate and ethyl octanoate. The grape components present in these fractions that are stimulating MCFA production may also be responsible for a requisite increase in the concentration of the analogous MCEE. It has been shown that increasing ethyl ester production in fermentations is more dependent on the concentration of the MCFA substrate than expression of the relevant yeast genes [26,27]. However, the large increases in MCEE concentrations in the wines made from the 80% acetone and 100% acetone/ethyl acetate fractions for both varietal series, predominantly observed for ethyl decanoate and ethyl dodecanoate (Figure 2), cannot be accounted for by MCFA content alone (Figure 3). As these volatiles were not present in these fractions before fermentation, the results imply that these non-polar fractions are either a source of unidentified precursors to MCEE or provide components that stimulate their production during fermentation.

Further fractionation of the CS100 fraction using diol-functionalised silica gel and fermentation of the resulting products identified several fractions that increased ethyl ester production during fermentation. The compounds present in one fraction were tentatively identified as the triacylglycerides TAG (18:2/18:2/18:2) and TAG (18:1/18:2/18:3). Subsequent experiments, where trilinolein (TAG (18:2/18:2/18:2)) was added to MGJM in increasing amounts before fermentation, showed that this triacylglyceride was able to stimulate the production of MCEEs (Figure 4). The shorter chain MCEE required higher concentrations of trilinolein to induce an increase in their concentrations compared to ethyl decanoate or ethyl dodecanoate, while ethyl decanoate required a higher concentration of trilinolein than ethyl dodecanoate did in order to see an increase in concentration in the final wine (Figure 4). Ethyl dodecanoate production was greatest after the addition of 0.34 g/L trilinolein, and when more trilinolein was added, the ethyl dodecanoate concentrations in the wines subsequently decreased, although they were still higher than the controls (Figure 4d). 

There are conflicting results concerning the effect of the addition of lipids to fermentations on the production of ethyl esters with some indicating that polyunsaturated fatty acids decrease MCEE production, while others indicate that they increase MCEE production. A study comparing fatty acids in grape musts with volatile compounds in wines found an inverse relationship between total initial linoleic acid content in grape must and final MCEE concentration in wine [28]. A similar finding was made when Sauvignon Blanc juice was supplemented with 131 mg/L linoleic acid, which decreased the concentration of most MCEE in the resulting wines [29]. In contrast, the addition of Tween80, which contains mostly oleic acid (70%) along with linolenic, palmitic and stearic acids (30% total), along with ergosterol to Chardonnay must resulted in the production of higher concentrations of ethyl hexanoate, octanoate and decanoate in the absence of oxygen addition [30]. This effect was more pronounced in a chemically-defined grape juice medium and extended to increased production of ethyl dodecanoate [30]. One issue with this study is that the changes in MCEE production may be attributed to the exogenous ergosterol and not just the added fatty acids. A more recent study has found that the addition of free oleic, linoleic and alpha-linolenic acids alters the volatile profile of yeast fermentations, with the concentrations of MCEE increasing in some of the fermentations [31]. The changes in ethyl ester production in response to the increasing concentration of the fatty acid mix was similar to that seen in this study (Figure 4), as a greater amount of the fatty acid mix was required to significantly increase ethyl hexanoate and octanoate concentrations in the wines than ethyl decanoate and dodecanoate [31]. However, these previous studies used free fatty acids compared to the triacylglyceride used in the experiments described in this manuscript. Recent lipidomic profiling of Sauvignon Blanc musts from New Zealand has found that free fatty acids represent less than 15% of the total lipids [32], and so, complex lipids could have a more important role than the free fatty acids during fermentation. Rosi and Bertuccioli [33] added a mixture of tripalmitin, triolein and trilinolein to synthetic medium, but found only minimal effects on ethyl ester production after fermentation. The insignificant effect may be because the concentration of the triacylglycerides added was approximately 20 mg/L total [33], which was below the lowest concentration used in this study.

The mechanism causing the increase in ethyl esters is not clear. It may, in fact, vary among the different ethyl esters quantified in the fermentations. For example, with regards to ethyl hexanoate, at least some of the increase in this ethyl ester after trilinolein addition appears to be due to chemical synthesis as some was formed in model wine spiked with 2.8 g/L trilinolein and incubated with the normal fermentations (Figure 4a). It is possible that the fatty acids from trilinolein are used as a substrate for beta-oxidation and the esters produced from esterification of the shorter chain products. However, a metabolic flux study suggested that ethyl hexanoate and ethyl octanoate originate from grape hexoses [34], and it is presumably the same for ethyl decanoate and dodecanoate. The increase in MCEEs could be due to some change in the number of fatty-acyl synthesis cycles that go to completion or the regulation of enzymes responsible for transforming acyl-CoA to MCEEs. This is in agreement with the data in Figure 4 as at lower trilinolein concentrations, the increase in the longer chain ethyl dodecanoate was greater than the shorter chain ethyl esters. As the concentration of trilinolein added to the must increased, the shorter chain ethyl esters were produced in greater amounts (Figure 4), suggesting that the fatty-acyl chains were being terminated after a lower number of cycles. However, the mechanism by which trilinolein might disrupt fatty acid synthase to release the MCFAs is unknown. If the acyl groups present in trilinolein are somehow transported into and accumulate in the yeast cells, this still does not agree with the model proposed by Dufour et al. [35] concerning the production of MCEEs, as the release of MCFAs was proposed to be due to the accumulation of saturated fatty acids. However, the model may only be taking into account endogenous unsaturated fatty acids, which in *Saccharomyces cerevisiae* are monounsaturated [36], and perhaps, linoleic acid can enhance the early release of the acyl chain from fatty acid synthase. Another possible mechanism by which MCEEs may accumulate in wine is if the incorporation of polyunsaturated fatty acids into yeast membranes causes the leakage of MCEE through the membrane into the media. However, the changes in the concentrations of the different ethyl esters would probably be expected to be linear in relation to the amount of unsaturated fatty acid in the medium if this were the case, which is not what was observed (Figure 4). It has also been suggested that unsaturated fatty acids and medium chain fatty acids perform a similar function in maintaining the fluidity of yeast membranes [37]. Therefore, if the cell can meet these needs with exogenous unsaturated fatty acids, then the medium chain fatty acids that are produced will be surplus to the requirements and may require removal in the form of volatile esters to avoid the disruption of cellular processes by the surfactant-like, free MCFA.

## 4. Materials and Methods

### 4.1. Chemicals and Materials

Solvents purchased from Merck (Sydney, Australia) were chromatographic quality. Diaion HP-20 poly(styrene-divinylbenzene) and diol-functionalised spherical silica gel (40–75-mm particle size, 100 Å pore size) were purchased from Supelco (Sigma, Sydney, Australia). All the chemical standards used to confirm compound identity and in calibration curves were purchased from Sigma with the exception of dodecanoic acid, which was obtained from Honeywell Fluka (Morris Plains, NJ, USA). The deuterated standards were purchased from C/D/N Isotopes (Pointe-Claire, Canada), with the exception of *d*_9_-ethyl nonanoate, which was synthesised as described in Boss et al. [7]. Trilinolein was obtained from Nu-Chek Prep Inc. (Elysian, MN, USA).

### 4.2. Grape Materials

Cabernet Sauvignon grapes were harvested from Willunga, South Australia, Australia, in March 2010, and Riesling grapes were harvested from Charleston, Adelaide Hills, South Australia, Australia, in March 2010. After transportation, bunches were stored overnight at 4 °C before the berries were removed from bunches and flash frozen in liquid nitrogen. Frozen berries were stored at −40 °C until further use.

### 4.3. Grape Fractionation

#### 4.3.1. Extraction of Grape Samples

A 1.35-kg sample of frozen Cabernet Sauvignon berries was crushed in an industrial 5-L blender. Acetone (500 mL) was added to the resulting pulp and extracted for 24 h at room temperature (~22 °C). The supernatant was filtered through Whatman Filter Paper 1 (Sigma) and stored in the dark at room temperature (Extract 1). The remaining solids were further extracted for 24 h with acetone (250 mL) and then filtered as above. The supernatant was collected and stored (Extract 2). A final acetone extraction (250 mL) was conducted on the remaining solids for 24 h. The supernatant was collected by filtration and stored (Extract 3). Frozen Riesling berries (~1.35 kg) were extracted by the above process to provide three acetone extracts for this cultivar.

#### 4.3.2. PSDVB Loading and Fractionation of Grape Extracts

Diaion HP-20 beads (~250 mL) were loaded into a glass chromatography column, then sequentially equilibrated with water, methanol and acetone (800 mL of each). Cabernet Sauvignon Extracts 2 and 3 were combined and passed through the column. The collected eluent was diluted with water (500 mL) to approximately 50% aqueous acetone. This was passed through the column again, and the collected eluent was further diluted to ~25% aqueous acetone. Extract 1 was diluted to ~25% aqueous acetone and was combined with the diluted Extracts 2 and 3. This solution was passed through the column again. The eluent was diluted to ~10% aqueous acetone and passed through the column one final time. The column was then washed with water (800 mL, discarded). Chromatography was performed by flushing the column with mixtures of increasing concentrations of acetone in water (800 mL each) to give fraction CS20 (Cabernet Sauvignon, eluted with acetone:water, 20:80, *v*/*v*; yield = 34.0%), fraction CS40 (40:60, *v*/*v*; yield = 45.9%), fraction CS60 (60:40, *v*/*v*; yield = 7.6%), fraction CS80 (80:20, *v*/*v*; yield = 6.3%), fraction CSA (100:0, *v*/*v*; combined yield with CSEA = 6.2%), and fraction CSEA (100% ethyl acetate). Subsamples of CSA and CSEA were combined and designated as CS100 for screening purposes. The Riesling extracts were loaded and fractionated according to the above procedure to provide 5 distinct Riesling grape fractions: R20 (Riesling, eluted with acetone:water, 20:80, *v*/*v*; yield = 18.0%), fraction R40 (40:60, *v*/*v*; yield = 54.6%), fraction R60 (60:40, *v*/*v*; yield = 12.7%), fraction R80 (80:20, *v*/*v*; yield = 6.5%), fraction RA (100:0, *v*/*v*; combined yield with REA = 8.2%), and fraction REA (100% ethyl acetate). Subsamples of RA and REA were combined and designated as R100.

#### 4.3.3. Diol Loading and Second Generation Fractionation of Grape Extract Fractions

Diol-functionalised silica gel (~20 mL each) was loaded in slurries with *n*-hexane into two glass chromatography columns, labelled ‘Column A’ and ‘Column B’, then each column was sequentially equilibrated with ethyl acetate (EtOAc), acetonitrile (MeCN) and *n*-hexane (60 mL of each). The acetone and ethyl acetate fractions, which together constituted the CS100 fraction, were respectively loaded onto either Column A or B in *n*-hexane. Chromatography was performed by sequentially flushing the column with ethyl acetate/hexane mixtures (60 mL each), then with acetonitrile/ethyl acetate mixtures (60 mL each), followed by methanol (MeOH; 60 mL) to give fractions A1 and B1 (100% *n*-hexane; yield = 0.0 and 11.7%, respectively), fractions A2/B2 (5:95, *v*/*v*, EtOAc:*n*-hexane; yield = 11.7 and 0.0%), fractions A3/B3 (10:90, *v*/*v*; yield = 20.2 and 4.6%), fractions A4/B4 (15:85, *v*/*v*; yield = 9.2 and 7.2%), fractions A5/B5 (20:80, *v*/*v*; yield = 8.1 and 7.1%), fractions A6/B6 (25:75, *v*/*v*; yield = 6.3 and 8.1%), fractions A7/B7 (30:70, *v*/*v*; yield = 3.8 and 0.0%), fractions A8/B8 (50:50, *v*/*v*; yield = 7.0 and 9.0%), fractions A9/B9 (100:0, *v*/*v*; yield = 4.6 and 9.5%), fractions A10/B10 (5:95, *v*/*v*, MeCN:EtOAc; yield = 1.6 and 3.8%), fractions A11/B11 (10:90, *v*/*v*; yield = 8.3 and 2.6%), fractions A12/B12 (15:85, *v*/*v*; yield = 0.4 and 25.0%), fractions A13/B13 (20:80, *v*/*v*; yield = 4.6 and 1.1%), fractions A14/B14 (25:75, *v*/*v*; yield = 9.0 and 0.7%), fractions A15/B15 (30:70, *v*/*v*; yield = 2.4 and 0.7%), fractions A16/B16 (50:50, *v*/*v*; yield = 0.2 and 1.1%), fractions A17/B17 (100:0, *v*/*v*; yield = 0.1 and 0.6%) and fractions A18/B18 (100, *v*/*v* MeOH; yield = 2.7 and 3.3%). The fractions were collected individually, separated from the solvent under vacuum and freeze-dried.

#### 4.3.4. NMR Spectroscopy

^1^H, ^13^C, COSY, HSQC and HMBC spectra were collected on a 600-MHz Bruker NMR spectrometer using deuterated chloroform as a solvent and referenced internally to the residual solvent signal (δ_C_ = 77; δ_H_ = 7.26) in a 5 mm-diameter NMR tube (Wilmad, Sigma).

#### 4.3.5. High-Resolution Mass Spectrometry

High-resolution mass spectrometry was performed on a Waters Synapt HDMS in positive ion mode across a mass range of 100–1000 *m*/*z*. Electrospray ionization was used with infusion. The sodium adduct of raffinose at *m*/*z* 527.1588 was used for a lock mass signal.

#### 4.3.6. LC-MS-MS

The liquid chromatography was carried out in an LC30AD (Shimadzu) with an Ascentis C18 column (Sigma Aldrich, St. Louis, MI, USA) with dimensions 2.1 × 15 mm and a 2.7-µm layer thickness. The column was held at 35 °C with a CTO-20A column oven (Shimadzu), with an elution profile, using two mobile phases, modified from the method of Bird and co-workers [38]. Mobile Phase A contained 40% acetonitrile and 60% water, with a total concentration of 7.5 mM ammonium formate. Mobile Phase B contained 10% acetonitrile and 90% isopropanol and also had a total concentration of 7.5 mM ammonium formate. The elution profile was: 1.5 min at 32% B, then increasing linearly to 45% B over 2.5 min, then to 52% B over 1 min, followed by an increase to 58% B over three min. The elution solvent was composed of 66% B by 11 min and 70% B three min later, followed by a gradient increasing to 75% over four min. In a departure from the method of Bird, the elution profile was extended and the gradient flattened after this, increasing to 97% B at 31 min and held at 97% B for four min. The flow-rate was 260 µL·min^−1^. Positive-ion mode ESI MS-MS spectra were collected on an ABSciex QTOF 5600 mass spectrometer with DuoSpray Ion Source operating at 550 °C. A TOF scan cycle was used to screen for precursor *m*/*z* candidates for MS-MS experiments (1700-ms scan cycle from *m*/*z* 100–1200 with a collision energy of 10 eV). The information-dependent acquisition mode was used with an *m*/*z* threshold of 200 counts per second within 50 mDa *m*/*z* in the TOF scan. Precursor *m*/*z* with intensities above this threshold would trigger the collection of MS-MS spectra centred on the triggering precursor *m*/*z*. The MS-MS spectra were collected with a collision energy of 30 eV with a collision energy spread of 15 eV and fragments collected from *m*/*z* 50–1200 with a pulser frequency of 16.913 kHz.

### 4.4. Model Must Fermentations

#### 4.4.1. Model Grape Juice Must Preparation

Model grape juice medium (MGJM) was prepared based on the protocol reported by Keyzers and Boss [9] with slight modifications. d-Glucose (120 g), d-fructose (120 g), 5 g d/l-malic acid, 5 g tartaric acid, 0.2 g citric acid, 1.7 g yeast nitrogen base (YNB) without ammonium sulphate (1000 mg·L^−1^ KH_2_PO_4_, 2 mg·L^−1^ myo-inositol, 0.04 mg·L^−1^ CuSO_4_, 500 mg·L^−1^ MgSO_4_, 0.4 mg·L^−1^ niacin, 0.1 mg·L^−1^ KI, 100 mg·L^−1^ NaCl, 0.2 mg·L^−1^ para-aminobenzoic acid, 0.2 mg·L^−1^ FeCl_3_, 100 mg·L^−1^ CaCl_2_, 0.4 mg·L^−1^ pyridoxine, 0.4 mg·L^−1^ MnSO_4_, 0.002 mg·L^−1^ biotin, 0.2 mg·L^−1^ riboflavin, 0.2 mg·L^−1^ Na_2_MoO_4_, 0.4 mg·L^−1^ calcium pantothenate, 0.4 mg·L^−1^ thiamine, 0.4 mg·L^−1^ ZnSO_4_, 0.002 mg·L^−1^ folic acid, 0.5 mg·L^−1^ H_3_BO_3_; MP Biomedicals (Santa Ana, CA, USA), 8 g Synthetic Complete (Hopkins) amino acid supplement mixture (684.8 mg·L^−1^ l-leucine; 342.4 mg·L^−1^ of the other 19 proteinogenic amino acids; 342.4 mg·L^−1^ myo-inositol; 342.4 mg·L^−1^ uracil; 84 mg·L^−1^ adenine and; 34.4 mg·L^−1^ para-aminobenzoic acid) and 0.3 g NH_4_Cl were dissolved in 1 L water. The pH of the resulting medium was adjusted to 3.2 by addition of KOH. The synthetic medium was sterilised by filtration (0.20-µm disposable sterile filter units, Nalgene, Rochester, NY, USA) prior to use.

#### 4.4.2. Yeast

Yeast starter cultures were prepared by adding ~0.25 g of yeast (strain EC1118, Prise de Mousse, ABMauri, Australia) to 25 mL of MGJM, which was incubated overnight at 28 °C with shaking. Prior to use, the starter culture was centrifuged (2500× *g* for 10 min), then re-suspended in 20 mL sterile water. This process was repeated a further two times. The starter culture was then diluted to 1.0 AU at 600 nm by dilution with sterile water for inoculation.

#### 4.4.3. Fermentation Conditions: Screen of First Round Fractionation

All fermentations (50 mL) were prepared under sterile conditions. A portion of each fraction (200 mL) was evaporated under reduced pressure to remove organic solvents and then freeze-dried to provide dry, solid subsamples of each fraction. These subsamples were dissolved in 12 mL sterile water/acetone (1:1, *v*/*v*) immediately prior to fermentation experiments. Fermentations were carried out by spiking 2 mL of the subsample of each fraction (CS20–CS100 and R20–R100) into the MGJM and then inoculated with yeast starter culture (1 mL), and controls contained the equivalent amount of solvent (sterile water/acetone 1:1) with no grape material. In doing this, the amount of grape material added to the must approximately equated to that extracted from 50 g of whole grape. Air-locks were used to maintain an anaerobic environment. In all cases, three separate ferments for each treatment were prepared by spiking the appropriate proportion of grape material to afford fermentation replicates. Fermentations were agitated twice daily and allowed to proceed until mass loss stabilised. Fermentation was halted by removing yeast cells by centrifugation (1250× *g* for 5 min). The clarified wines were then stored in glass at 4 °C overnight prior to analysis.

#### 4.4.4. Fermentation Conditions: Screen of Second Round Fractionation 

Due to the very limited material available after the second round of fractionation using the diol-functionalised silica gel, a very small-scale fermentation procedure was used for screening these fractions for their ability to increase ethyl ester production. These “micro-fermentations” (1 mL) were prepared by adding 1 mL of MGJM into 2-mL plastic microfuge tubes, each containing 1 mg of the diol fractions (A1–A18 or B1–B18) and inoculating with yeast starter culture (20 µL), while controls contained MGJM, yeast starter culture and no grape material. In each case, only one fermentation for each fraction was carried out, except for the control fermentation, which was carried out in triplicate. The lids of the microfuge tubes were pierced with a sterile needle so that pressure could be released from the tube. The fermentations were agitated twice daily and allowed to proceed until mass loss was stabilised. Fermentation was halted by removing yeast cells by centrifugation (2500× *g* for 5 min). The clarified wines were stored in new microfuge tubes at 4 °C overnight prior to analysis. 

### 4.5. Analysis of Volatile Compounds

#### 4.5.1. Volatile Analysis of Wines 

Solid phase microextraction-gas chromatography mass spectrometry (SPME-GCMS) was used to analyse the volatile constituents of the wines produced from the fermentation of the spiked MGJM mixtures. Aliquots of the wines (5 mL) were diluted 1 in 2 with H_2_O to a final volume of 10 mL before analysis. In all cases, NaCl (3 g) was added to each SPME vial (amber 20 mL, Supelco) prior to sample addition. Samples were spiked prior to analysis with 10 µL of a solution containing the following deuterated internal standards at the specified concentrations: *d*_13_-hexanol (920 mg/L); *d*_11_-hexanoic acid (930 mg/L); *d*_16-_octanal (82.1 mg/L); *d*_5_-ethyl nonanoate (9.2 mg/L), *d*_3_-linalool (1.73 mg/L). SPME-GCMS was carried out using an Agilent 7890A gas chromatograph (Santa Clara, CA, USA) equipped with a GerstelMP2 auto-sampler and using an Agilent Technologies 5975C mass spectrometer for detection of *m*/*z* and compound identification. SPME and chromatography conditions and compound identification were carried out as reported by Dennis et al. [8]. Quantification of the analytes was achieved by linear 5-point external calibrations (r^2^ = 0.97–0.99). Standard compounds were added to model wine (12% aqueous ethanol, pH adjusted to 3.2 with potassium bitartrate) at five different concentrations, and further preparation of the SPME vials was analogous to the above procedure for samples.

#### 4.5.2. Volatile Analysis of Fractionated Grape Extract Samples

To test for the presence of any wine volatiles released without fermentation from the non-volatile fractionated grape extracts, SPME-GCMS was used. For each fraction (R20–R100 and CS20–CS100), 200 µL of each re-dissolved subsample were spiked into 5 mL of model wine (12% EtOH (*v*/*v*) with 2 g·L^−1^ potassium hydrogen tartrate, pH 3.69) and diluted 1 in 2 with H_2_O to a final volume of 10 mL before analysis. In all cases, NaCl (3 g) was added to each SPME vial (20 mL) prior to sample addition. Samples were spiked with *d*_13_-hexanol as an internal standard (10 µL of 920 mg·L^−1^) prior to SPME-GCMS analysis. SPME-GCMS analysis was carried out as described above for the analysis of wine volatiles.

#### 4.5.3. Headspace Volatile Analysis of Micro-Fermentation Wines

SPME-GCMS was used to analyse the volatile constituents of wines produced by micro-fermentation of spiked MGJM. Aliquots of 1 mL of wine were added to 0.3 g NaCl in each SPME vial (10 mL). Samples were spiked with 10 µL of methanolic solution containing 920 mg·L^−1^ *d*_13_-hexanol, 930 mg·L^−1^ *d*_11_-hexanoic acid and 6.12 µg·L^−1^ methyl nonanoate. SPME and chromatography conditions were otherwise carried out as for the wine samples in the previous section, as reported by Dennis et al. [8].

#### 4.5.4. Data Analysis

The effect of adding separate grape fractions to the MGJM on the concentration of volatiles in the headspace of the wines was analysed by one-way ANOVA using SPSS 16.0 (SPSS Inc., Chicago, IL, USA). When the mean peak areas of volatile compounds were found to be significantly different, Duncan’s post hoc tests were performed to determine significant differences (*p* < 0.05) among the fractions. Comparisons were only made amongst the different fractions of each variety, not between the varieties.

## 5. Conclusions

In summary, the utility of a natural products separation procedure involving cyclic loading (Supplementary Figure S2) has proven to be an effective means for fractionating grape berry extracts to identify grape compounds that can influence the production of the volatile components of wines. It was shown that the fractionation of grape acetone extracts and fermentation of the dried fractions can be used to confirm the presence of non-volatile precursors of wine volatile compounds in the grapes and identify fractions for subsequent chromatographic separation. This work also suggested that there may be inhibitors of yeast-derived wine volatile components in the grape fractions. A second round of separation using one of the initial fractions led to the identification of a subsequent fraction containing a mixture of two triacylglycerides that greatly increased the production of MCEEs in fermentations. This was confirmed by the fermentation of model musts spiked with increasing amounts of one of those triacylglycerides, trilinolein. The mechanism for this enhanced ethyl ester production is unknown and is the focus of current research.

## Supplementary Materials

The following are available online at www.mdpi.com/1420-3049/23/1/152/s1.

## Appendix A. Determination of Fraction B1 Composition

By means of 1D/2D NMR, HR-MS and LC-MS/MS experiments, the triacylglycerides TAG (18:2/18:2/18:2) and TAG (18:1/18:2/18:3) were confirmed in an approximate 1:1 ratio in fraction B1. TAG (18:2/18:2/18:2) and TAG (18:1/18:2/18:3): ^1^H-NMR (CDCl_3_, 600 MHz) δ 5.39 (dtt, *J* = 10.6, 7.2, 1.5 Hz, H-9′18:3 and H-16′18:3), 5.37 (dtd, *J* = 10.5, 7.3, 1.5 Hz, H-10′18:3, H-12′18:3, H-13′18:3, H-15′18:3), 5.35 (dt, *J* = 10.7, 6.8 Hz, H-10′18:2, H-12′18:2), 5.33 (dt, *J* = 10.7, 7.0 Hz, H-9′18:2, H-13′18:2), 5.30 (dtt, *J* = 10.4, 6.9, 1.5 Hz, H-9′18:1 and H-10′18:1), 5.26 (1H, tt, *J* = 5.9, 4.3 Hz, H-2), 4.29 (2H, dd, *J* = 11.9, 4.4 Hz, H-1a and H-3a), 4.14 (2H, dd, *J* = 11.9, 5.9 Hz, H-1b and H-3b), 2.80 (tt, *J* = 6.9, 1.5 Hz, H-11′18:3, H-14′18:3), 2.77 (2H, t, *J* = 6.8 Hz, H-11′18:2), 2.32 (t, *J* = 3.8 Hz, H-2′18:3), 2.31 (2H, t, *J* = 3.8 Hz, H-2′18:2), 2.30 (t, *J* = 3.8 Hz, H-2′18:1), 2.04 (4H, dt, *J* = 7.0, 6.9 Hz, H-8′18:2, H-14′18:2), 2.08 (dt, *J* = 7.5, 7.6 Hz, H-8′18:3, H-17′18:3), 2.01 (dtd, *J* = 6.8, 6.8, 1.5 Hz, H-8′18:1 and H-11′18:1), 1.57–1.64 (m, H-3′18:1, H-3′18:2, H-3′18:3), 1.23–1.38 (m, H-4′18:1, H-5′18:1, H-6′18:1, H-7′18:1, H-12′18:1, H-13′18:1, H-14′18:1, H-15′18:1, H-16′18:1, H-17′18:1, H-4′18:2, H-5′18:2, H-6′18:2, H-7′18:2, H-15′18:2, H-16′18:2, H-17′18:2, H-4′18:3, H-5′18:3, H-6′18:3, H-7′18:3), 0.97 (t, *J* = 7.6 Hz, H-18′18:3), 0.89 (t, *J* = 6.9 Hz, H-18′18:2), 0.87 (t, *J* = 7.0 Hz, H-18′18:1); ^13^C NMR (CDCl_3_, 150 MHz) δ 68.9 (CH, C-2), 62.1 (CH_2_, C-1 and C-3), 173.3 (C, C-1′18:1(sn-1,3)), 173.2 (C, C-1′18:2(sn-1,3)), 172.9 (C, C-1′18:1(sn-2) and C-1′18:2(sn-2)), 132.0 (CH, C-16′18:3), 130.2 (CH, C-13′18:2, C-9′18:3), 130.01 (CH, C-9′18:2), 129.98 (CH, C-10′18:1), 129.71 (CH, C-9′18:1(sn-1,3)), 129.68 (CH, C-9′18:1(sn-2)), 128.3 (CH, C-13′18:3), 128.2 (CH, C-12′18:3), 128.07 (CH, C-10′18:2(sn-2)), 128.05 (CH, C-10′18:2(sn-1,3)), 127.9 (CH, C-12′18:2(sn-1,3)), 127.8 (CH, C-10′18:3(sn-2)), 127.7 (CH, C-10′18:3(sn-1,3)), 127.1 (CH, C-15′18:3), 34.2 (CH_2_, C-2′18:1(sn-2), C-2′18:2 (sn-2) and C-2′18:3 (sn-2)), 34.04 (CH_2_, C-2′18:1(sn-1,3)), 34.02 (CH_2_, C-2′18:2(sn-1,3) and C-2′18:3(sn-1,3)), 29.77–29.04 (CH_2_, C-4′18:1–C-7′18:1, C-12′18:1–C-16′18:1, C-4′18:2–C-7′18:2, C-15′18:2–C-16′18:2 and C-4′18:3–C-7′18:3), 27.2 (CH_2_, C-8′18:1, C-11′18:1, C-8′18:2,C-14′18:2 and C-8′18:3), 25.6 (CH_2_, C-11′18:2, C-11′18:3 and C-14′18:3), 24.87–24.83 (CH_2_, C-3′18:1, C-3′18:2 and C-3′18:3), 22.7 (CH_2_, C-17′18:1), 22.6 (CH_2_, C-17′18:2), 20.6 (CH_2_, C-17′18:3), 14.3 (CH_3_, C-18′18:3), 14.13 (CH_3_, C-18′18:1), 14.09 (CH_3_, C-18′18:2); HR-MS (ESI+) *m*/*z* 879.7458 (calcd. for C_57_H_90_O_6_ +, 879.7442) and 901.7286 (calcd. for C_57_H_89_O_6_Na+, 901.7261); LC-TOF-MS (ESI+), *m*/*z* 896.76526 ([M + NH_4_]^+^), *m*/*z* 896.77017 ([M + NH_4_]^+^, calculated for [C_57_H_98_O_6_ + NH_4_]^+^); MS/MS (ESI+, +30 15 V), *m*/*z* (%) 599.5014 (100), 879.7415 (37), 597.4858 (27), 601.5173 (23).
