# Supplementary material for: Fermentation-Guided Natural Products Isolation of a Grape Berry Triacylglyceride that Enhances Ethyl Ester Production
Source: Molecules. 2018 Jan 12;23(1):152. doi: 10.3390/molecules23010152 (PMC6017315; doi:10.3390/molecules23010152)
Supplement: Supplementary file 1 [file molecules-23-00152-s001.docx]

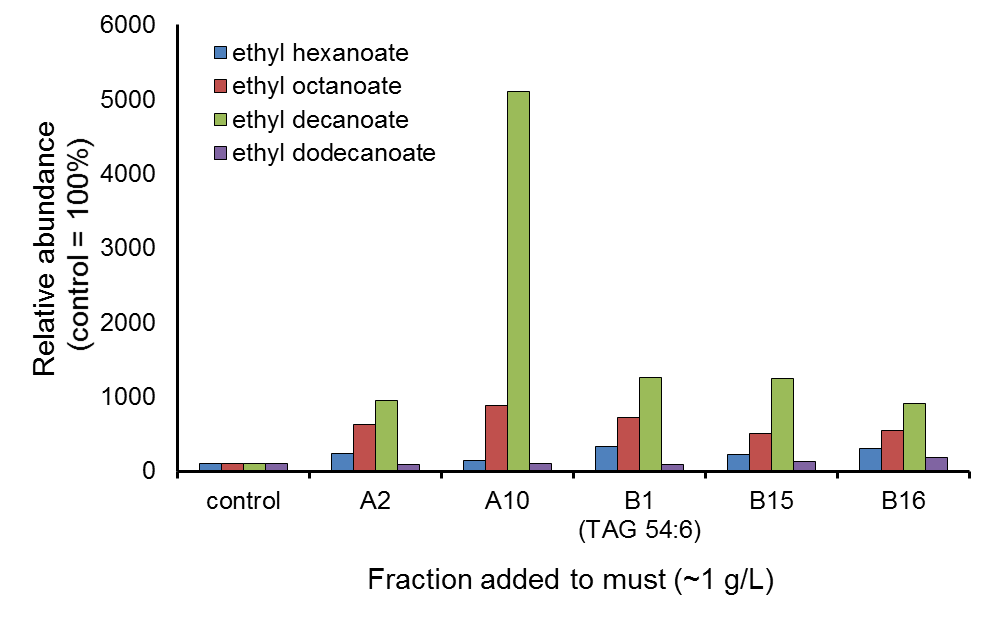


**Supplementary Figure 1.** Concentrations of MCEE in micro-wines prepared from musts supplemented with second generation fractions A2, A10, B1, B5 and B16 (n=1) or un-supplemented controls (n=3). Concentrations are presented as a percentage of the relative concentration in control wines fermented without supplements. Fraction B1 contained a mixture of two triacylglycerides each with the three acyl groups predicted to contain 54 carbons and 6 double bonds (TAG 54:6).

Grape acetone extract

Fractionation using HP-20 column

Five grape fractions

Fermented in model must & analysed by GCMS

Fraction selected that produced highest conc ethyl esters

2^nd^ round fractionation with Diol column using various solvents

Eighteen 2^nd^ round fractions

Fermented at very small scale & analysed by GCMS

Five selected fractions

NMR conducted on fractions, Look for “clean” proton spectrum

Selected fraction

LCMS/MS

HR-MS

Compound identified, activity in fermentation confirmed using commercial standard

**Supplementary Figure 2.** Diagram for the experimental workflow that led to the isolation of the triacylglyerides.
